# Epigenetic crosstalk between hypoxia and tumor driven by HIF regulation

**DOI:** 10.1186/s13046-020-01733-5

**Published:** 2020-10-27

**Authors:** Tiansheng Li, Chao Mao, Xiang Wang, Ying Shi, Yongguang Tao

**Affiliations:** 1 NHC Key Laboratory of Carcinogenesis and Hunan Key Laboratory of Translational Radiation Oncology, Hunan Cancer Hospital and The Affiliated Cancer Hospital of Xiangya School of Medicine, Central South University, Changsha, Hunan China; 2 Key Laboratory of Carcinogenesis and Cancer Invasion of the Chinese Ministry of Education, Cancer Research Institute, Central South University, Changsha, Hunan China; 3 Department of Thoracic Surgery, Second Xiangya Hospital, Central South University, Changsha, 410011 China

**Keywords:** Hypoxia, Hypoxia-inducible factors (HIFs), Tumors, Epigenetic regulation, Crosstalk, Therapeutic strategy

## Abstract

Hypoxia is the major influence factor in physiological and pathological courses which are mainly mediated by hypoxia-inducible factors (HIFs) in response to low oxygen tensions within solid tumors. Under normoxia, HIF signaling pathway is inhibited due to HIF-α subunits degradation. However, in hypoxic conditions, HIF-α is activated and stabilized, and HIF target genes are successively activated, resulting in a series of tumour-specific activities. The activation of HIFs, including HIF-1α, HIF-2α and HIF-3α, subsequently induce downstream target genes which leads to series of responses, the resulting abnormal processes or metabolites in turn affect HIFs stability. Given its functions in tumors progression, HIFs have been regarded as therapeutic targets for improved treatment efficacy. Epigenetics refers to alterations in gene expression that are stable between cell divisions, and sometimes between generations, but do not involve changes in the underlying DNA sequence of the organism. And with the development of research, epigenetic regulation has been found to play an important role in the development of tumors, which providing accumulating basic or clinical evidences for tumor treatments. Here, given how little has been reported about the overall association between hypoxic tumors and epigenetics, we made a more systematic review from epigenetic perspective in hope of helping others better understand hypoxia or HIF pathway, and providing more established and potential therapeutic strategies in tumors to facilitate epigenetic studies of tumors.

## Background

Low oxygen tension (hypoxia) arises from excessive oxygen consumption to supports the demand of rapid proliferation, and abnormalities in the structure and function of blood vessels within solid tumors [1–4]. Mounting clinical and experimental evidences have revealed that hypoxia-related oxygen pressure contributes to higher metastasis and mortality rates [5–7]. Hypoxia occurs in 90% of solid tumors, which has been regarded as a hallmark of cancer [8–10]. In addition, hypoxia often plays a key role in tumor progression and tolerance to targeted therapies [11, 12]. Massive efforts have been brought about in investigating hypoxia due to its significantly clinical implication.

Tumor-associated metabolic alterations at multi-steps of metastasis have been observed in clinical samples via ever-accelerated updating of molecular biological tools. Particularly, it has become evident that adaptation in metabolite-driven gene regulation may be a potent hallmark to measure tumorigenesis [13–15]. Hypoxia-inducible factors (HIFs) are heterodimers composed of α subunits and β subunits, where α subunits include HIF-1α, HIF-2α, and the less studied HIF-3α [12, 16, 17]. Under normoxia, two prolines residues of HIF-1α and HIF-2α are hydroxylated by prolyl hydroxylase domain protein 2 (PHD2) and go through ubiquitin-mediated proteolysis via binding to Hippel-Lindau tumor suppressor (VHL) [18]. However, these ubiquitination processes are inhibited due to enzyme inactivation within solid tumors, which leading to accumulating HIF-1α and HIF-2α stability [19]. HIF-1, existing in the form of functional heterodimer which consisting of α and β (aryl hydrocarbon receptor nuclear translocator, ARNT) isoforms, is a primary sensor of oxygen limitation and its induction supports cancer cells proliferation during hypoxia by eliciting several metabolic alterations [4, 20]. Likely, HIF-2α/ARNT heterodimer, known as HIF-2, is sensible to oxygen availability in tumors, and is also tightly controlled by proteasomal degradation via prolyl hydroxylases (PHDs) in both normoxic and hypoxic conditions [21]. Moreover, HIF-2α promotes tumor progression via macrophage lactate /HIF-2α/ATP6v0d2 axis [22], and some lncRNAs may be its transcriptional targets within solid tumors [21]. Role of HIF-3α has not yet been fully understood, which has long been thought negatively associated with HIF-1α and HIF-2α expression and function to directly or indirectly regulate hypoxia-induced pathological processes [23–25]. For example, TIMP2 (tissue inhibitor of metalloproteinases 2) blockade by HIF-1α/miR-210/HIF-3α feed circuit often plays a significantly role in regulating hepatocellular carcinoma (HCC) metastasis, which is regard as associated with poor prognostic effects [26].

HIF signaling directly or indirectly get a tightly command of physiological and pathological functions of numerous genes associated with carcinogenesis mechanisms, which refer to the regulation of proliferation, cell death, radiotherapy and chemotherapy [24, 25, 27–30], tumor microenvironment [31–33], metastasis [34–36], angiogenesis [37–40], and metabolic reprogramming [41–43] etc. within solid tumors. Epigenetics refers to a heritable change in gene expression when DNA sequence is not changed, that is, the genotype is not changed but the phenotype is changed [44, 45]. It’s an out-of-sequence form of inheritance. In addition to the genetic information provided by DNA sequence, epigenetic information provides instructions on when, where and how to apply genetic information such as DNA methylation, histone modification, and nucleosome positioning [44]. Epigenetic research has been an important part of cancer research providing accumulating basic or clinical evidences for tumor treatments [46, 47]. In the present review, we put our eyes on the role of HIF signaling and hypoxia-dependent regulator in tumor progression from the perspective of epigenetics.

## Canonical and non-canonical regulation of HIF signaling

**Fig. 1:**
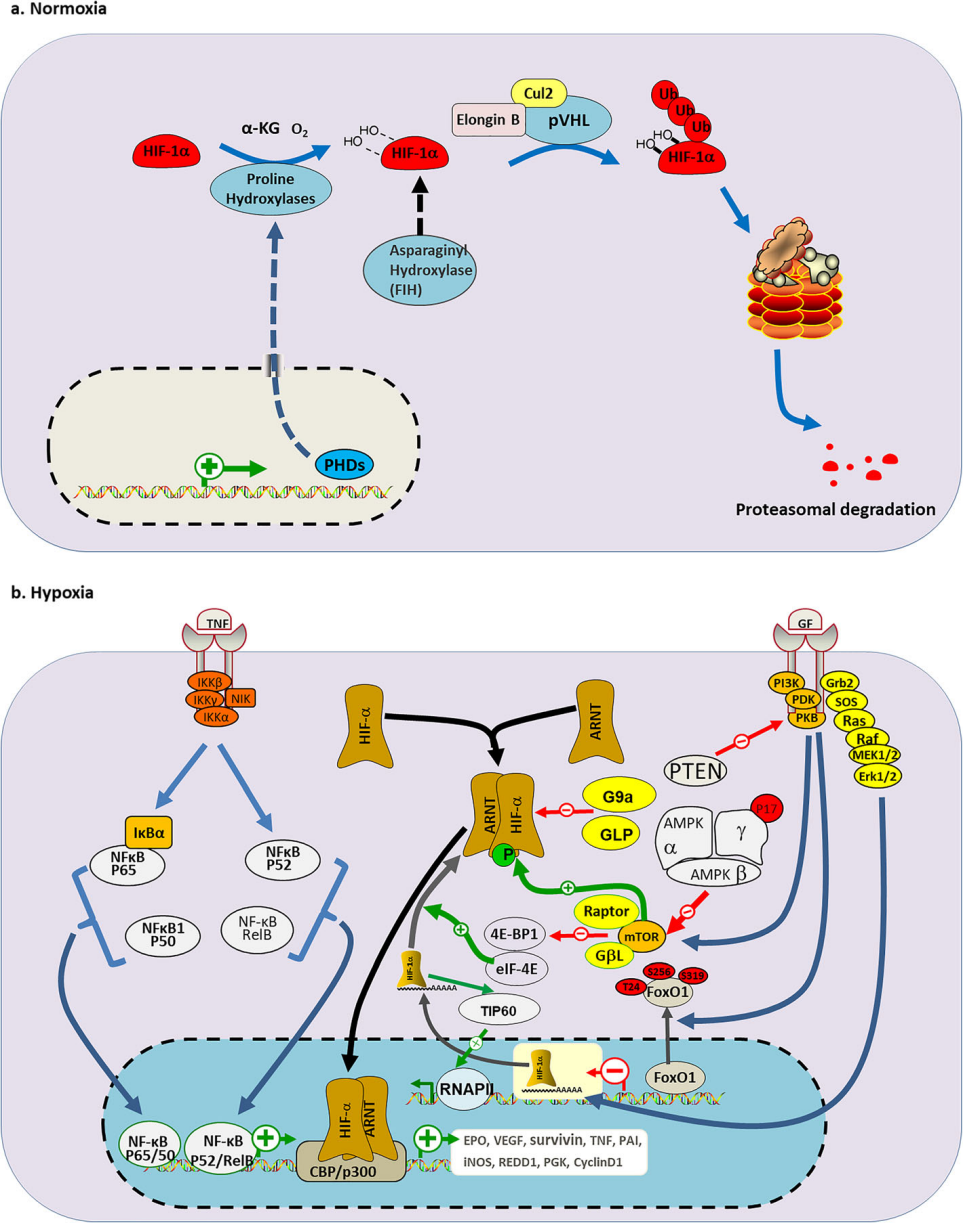
Canonical and non-canonical regulation of HIF signaling. **a** Oxygen- dependent regulation of HIF-α. Under normoxic condition, the ODD module within HIF-1α will be degraded via binding to VHL E3 ubiquitin ligase complex which consisting of pVHL, Cullin 2 (Cul-2) and Elongin B. This process is mediated by ubiquitin-proteasome pathway, which α-ketoglutarate-dependent PHDs catalyze the hydroxylation of ODD domain which is recognized by VHL, eventually leading to proteasomal degradation of HIF-α. More, factor inhibiting HIF (FIH) inhibits the binding of p300 to HIF-α by hydroxylating asparagine residue within C terminal domain, which play a role of inhibition on HIF-α activity. **b** Regulation of the HIF pathway at mRNA and protein level. In hypoxic conditions, inhibition of PHDs promote the heterodimer formation consisting of HIF-α and ARNT. Extracellular signaling TNF-α stimulates I-κB kinase (IKK) complex which is comprised of IKKα and IKKβ, and other normal TNF signaling (NIK), which contribute to p65/50 complex and p52/RelB complex formation. Many other components NF-κB together activate target genes, including HIF-α, and further induce inflammation. More, PI3K, PDK and PKB activation induced by growth factors (GFs) activates mTOR pathway results in elevated HIF-α transcriptional activity. And phosphorylation of FoxO1 PI3K/PKB, which is transferred from the nucleus to the cytoplasm, prevents FoxO1 from acting on HIF-α. G9a/GLP methylates HIF-1α protein and inhibits HIF-1α activity within solid tumors, making it unable to bind to the hypoxic response element (HRE) of its target genes, resulting in inhibition of the downstream HIF pathway. More, HIF-1α acts on TIP60, which leads to chromatin histone acetylation and then to the activation of polymerase II, which ultimately activates HIF-1α target genes transcription. IκB, nuclear factor of κB inhibitor, alpha; IKK, IκB kinase; 4EBP1, eukaryotic translation initiation factor 4E-binding protein 1; eIF-4E, eukaryotic translation initiation factor; GβL: G protein beta subunit-like; Grb2: growth factor receptor-bound protein 2; EPO: erythropoietin; PAI: plasminogen activator inhibitor; iNOS: nitric oxide synthase; REDD1: regulated in development and DNA damage response 1; PGK: phosphoglycerate kinase

HIF family mainly mediates cellular oxygen tension-dependent reactions via a basic helix-loop-helix structure with a significant implication in pathological processes in tumors [4, 48]. It’s reported that HIF-1α and HIF-2α are widely expressed in various cell types and special tissues, respectively [49]. In the state of canonical regulation, HIF signaling is activated transcriptionally by the binding of HIF-1α or HIF-2α to their selective binding partner HIF-1β [4, 11]. In the normoxic case, the oxygen-dependent degradation (ODD) domain within HIF-1α confers instability for HIF-α function [50]. The ODD module will be degraded via binding with VHL which playing a role of E3 ubiquitin ligase complex in part through ubiquitin-proteasome pathway. In detail, prolyl hydroxylases (PHDs) catalyze the hydroxylation of ODD domain which is recognized by VHL, eventually leading to proteasomal degradation of HIF-α (Fig. 1a) [51]. With that in mind, inactivation of VHL, including mutations and other modifications such as methylation, is associated with various illnesses such as clear-cell renal cell carcinoma (ccRCC) due to aberrant VHL/HIF axis and may also affects human phenotypes [52, 53]. VHL encodes two RNA (variant 1 and variant 2), three different protein isoforms (pVHL213, pVHL160 and pVHL172). The pVHL213 and pVHL160 is translated from variant 1 through alternative splicing, and pVHL172 from variant 2. Recently, relevant studies revealed that pVHL172 is not involved in HIF signaling other than pVHL213 and pVHL160 [54, 55]. Oxygen concentration-dependent mechanism involving PHDs, exhibits a canonical example to perform response to hypoxia. In addition, under normoxia, an asparagine residue in the C-terminal activation domain of HIF-1α and HIF-2α is hydroxylated by factor inhibiting HIF (FIH), resulting in the inability of the region to bind to p300, thus weakening the activation of the HIF pathway. However, in hypoxia, due to the inactivation of FIH, HIF-1α and HIF-2α avoid being hydroxylated, and subsequently translocate into the nucleus to bind with ARNT and p300, leading to the activation of HIF target genes [56]. The effect of FIH on the HIF-α further deepens our understanding of the mechanisms governing the stability of HIF-α.

However, in mionectic cells, including hypoxic tumor cells and stromal cells, condition is characterized by non-canonical signaling pathway [11]. Metabolic reprogramming of tumor cells is characterized by the balance of glycolysis and oxidative phosphorylation (OXPHOS), and the interaction between the crosstalk and HIF signaling, especially HIFs stability is largely unclear [57]. OXPHOS damage is ever known to promote HIF-1α stabilization in normoxia and hypoxia, however, a series of studies noted that OXPHOS damage accompanied with mitochondrial complex I (CI) dysfunction may reduce HIF-1α activity, and glycolysis reduced by 5′ AMP-activated protein kinase (AMPK) is negatively associated with HIF-1α activity due to ATP supply and reactive oxygen species (ROS) production which promotes tumor progression [58–61]. Besides the regulation of OXPHOS genes, HIF-1 may be affected by many other factors, including hormones, growth factors and cytokines etc. on transcriptional and post-transcriptional level [62]. Increasing studies have elucidated that HIF genes could be regulated in the NF-κB (nuclear factor kappa-B) dependent manner. Some think that NF-κB pathway is activated by TNF-α (tumor necrosis factor alpha) when relevant components of this pathway translocated into nucleus, and subsequent changes are performed on HIF-1α level [63–65]. Others demonstrated that HIF-1β would be modulated directly by TNF-α after the adaptation of α subunit, all this finally led to changed HIF-2α gene expression in the presence of inflammatory cytokines, such as tat-associated kinase complex catalytic subunit (TAK), I-kappaB kinase (IKK) and cyclin-dependent kinase 6 (CDK6) [65–67]. More interestingly, authentic study has noted that TNFSF14 (tumor necrosis factor superfamily, member 14), as a non-canonical inducer of NF-κB, could perform a direct alteration on HIF-2α expression and activity. Mechanistically, TNFSF14-induced p52 selectively binds with certain sites of HIF-2α promoter leads to elevation of HIF-2α expression [68] v. In addition to the TNF signaling, PI3K/Akt/mTOR pathway also play a significant role in regulating HIFs activity, for example, phosphoinositide 3-kinase (PI3K), pyruvate dehydrogenase kinase (PDK) and protein kinase B (PKB) activation induced by growth factors activate mammalian target of rapamycin (mTOR) pathway results in elevated HIF-α transcriptional activity. And phosphorylation of forkhead box protein O1 (FoxO1), which is transferred from the nucleus to the cytoplasm, prevents FoxO1 from acting on HIF-α (Fig. 1b) [19, 69]. Recent studies have shown that up-regulated expression of interleukin-1β (IL-1β) secreted by B cells promotes the activation of HIF-2α, and HIF-2α activation further promotes the activation of delta like canonical Notch ligand 4 (DLL4) signaling, and finally interact with neurogenic locus notch homolog (Notch) to form the IL-1β/HIF-2α/Notch 1 axis [70]. Collectively, various mechanisms drive HIF signaling activation in low oxygen tensions to maintain the state of various cancer cell types, and TNF-α interacts with HIF signaling provides a preferable and typical example.

## Epigenetic regulation of HIFs activity

Growing evidence has revealed that the transcriptional activity of HIFs is regulated by epigenetic factors at multiple levels [71, 72]. The stability of HIF-α subunits are the basis for biological function of HIF complexes and regulates hypoxia-related phenotypes of tumor cells in combination with other important epigenetic regulators [73]. Lots of different epigenetic factors, including enzymes that play a role in methylation and acetylation, and non-coding RNAs, are closely related to HIFs stability and transcriptional activity [74].

### Effects of DNA methylation and demethylases on HIF-α activity

DNA methylation is an important event of epigenetic regulation within hypoxic tumors [45]. DNA methylation is a process in which S-adenosylmethionine (SAM) is used as a methyl donor to transfer methyl groups to specific bases under the catalysis of DNA methyltransferase [75, 76]. But in human cancers, DNA methylation occurs mainly at cytosine of CpG sites to produce 5-methylcytosine (5mC) [77, 78]. DNA methylation can regulate HIFs stability, and subsequently influence HIFs target genes expression [2]. Among them, promoter hypermethylation could lead to tumor suppressor genes silencing in cancer [79]. For instance, VHL hypermethylation at promoter site increases transcriptional activation of HIF-1α and promotes HIF-1α target gene activation such as carbonic anhydrase 9 (CA9) and glucose transporter type 1 (GLUT1) [2, 80]. DNA demethylation is also associated with dynamic regulation of HIF-1α. For example, HIF-1α promoter is demethylated at CpG site within colon cancer, which promoting the binding of HIF-1α protein to its own promoter and thus affecting HIF-1α transcriptional activation and target genes activation [2, 81]. DNA demethylation mediated by ten-eleven-translocation 5-methylcytosine dioxygenases (TET) plays an important role in regulating hypoxia-induced transcriptional program [82]. The 2-oxoglutarate-dependent dioxygenases (2-OGDDs) is a large family of about 70 members, of which the TET1-3 proteins are important members [83]. The TET proteins has the function of hydroxylation, which converts the 5mC in DNA to 5-hydroxymethylcytosine (5hmC), 5-formylcytocine, and 5-carboxylcytocine, leading to DNA demethylation in the consecutive biochemical reactions [84, 85]. However, it is the protein-protein interaction between TETs and other proteins, rather than the dependence of TET’s demethylase activity, that regulates the functional activity of HIF-α [75]. For example, 5hmC mediated by TET1 enzyme has been regarded as an important epigenetic DNA modification in brain, and can interacts with HIF-1α protein to regulate the responses induced by chronic restraint stress (CRS) in mice with CRS-induced depressive phenotype [86, 87]. Cheng et al. found that deletion of the TET1 gene resulted in CRS resistance in mice, conversely, and that the stress-induced hydroxymethylated loci (SI-DhMLs) were enriched with HIF-1α binding regions via genome-wide profiling [86]. Then, they identified that the elevated HIF-1α binding under CRS is related to SI-DhMLs through biochemical and chromatin immunoprecipitation sequencing (ChIP-seq) [87]. Together, these results shows that TET1 enzyme regulates stress-induced response by interacting with HIF-1α protein [87]. These results suggest that TET1 can regulate biochemical reactions by interacting with HIF-1α, rather than directly depending on DNA demethylase activity of TET1 on HIF-1α in non-tumor cells [87]. The interaction of TET1 with HIF-1α has also been further confirmed in neuroblastoma [88]. Hypoxia elevates global 5hmC level in DNA, and high level of 5hmC is closely related to the active expression of hypoxia-responsive target genes [82]. Part of the 5hmC was colocated with the hypoxic response element (HRE), promoting DNA demethylation and HIF binding [2]. Hypoxia leads to transcriptional activation of TET1 in HIF-1 dependent manner, and TET1 enzyme increases global 5hmC level [88]. These results suggest that TET1-mediated 5hmC alterations play an important role in the hypoxic response via HIF-1 binding rather than DNA demethylation of HIF-1α [88].

### The m^6^A methylation of mRNA involves in epigenetic regulation of HIF-α activity

To some extent, N6-methyladenosine (m^6^A) methylation can also regulate the stability of HIF-α within tumor cells. The m^6^A methylation on the 6th position of RNA molecule adenine, is the most common post-transcriptional modification of eukaryotic mRNA, occurring in about 25% of transcripts at genomic level [89–91]. For example, affects the methylation status of HIF-2α mRNA. As the m^6^A site identifier and RNA-binding protein, MTHFD2 is mainly involved in specific recognition of m^6^A-modified mRNAs in the cytoplasm, including m^6^A methylation of HIF-2α mRNA [92, 93]. MTHFD2 leads to increased stability of HIF-2α mRNA, which further improve HIF-2α translation [92]. Increased HIF-2α translation, in turn, elevates the transcriptional activity of MTHFD2 and finally results in a series of metabolic alterations [92]. This suggests that other m6A methylases may also be related to the stability of HIFs, but the epigenetic mechanisms remain to be further studied.

### Histone methylation and demethylation affect HIF-α activity

At present, histone methylation has been gradually recognized as an important regulatory factor driving malignant transformation of hypoxic tumors [94]. For example, G9a and G9a-like protein (GLP), as histone lysine methylases, regulate HIF-1α transcriptional activity and drive hypoxic-induced genes regression (Fig. 1b) [94, 95]. G9a, encoded by euchromatic histone lysine methyltransferase 2 (Ehmt2) mRNA, is an epigenetic regulator that methylates histone H3 lysine 9 (H3K9) and leads to condensed chromatin [96]. Since G9 is genetically down-regulated in a variety of tumors and can inhibit the expression of tumor suppressor genes, it plays an important role in carcinogenesis [2, 95]. The stability of G9a protein is increased due to the reduction of prolyl hydroxylation, which reducing the interaction between G9a and pVHL and subsequent proteasomal degradation [95]. A subset of genes which are necessary for hypoxic tumor suppression, are repressed due to increased H3K9 methylation by G9a [95]. And an G9a inhibitor, BIX01294, decreases the levels of PHD2, pVHL and vascular endothelial growth factor (VEGF), leading to increased stability of HIF-1α protein and reduced angiogenic activity [97]. However, the direct regulatory effects of G9a on HIF-1α and even HIF-2α remain unclear. GLP generally plays a synergistic epigenetic modification function with G9a in tumors [94, 98]. For example, H3K9 methylation mediated by G9a and GPL enzymes depends on the functional activity of FIH. Under normoxic conditions, G9a and GLP were hydroxylated by FIH at the Asn779 and Asn867, respectively. After hydroxylation, G9a and GLP lost the function of methylating H3K9 [99]. Under hypoxic conditions, G9a and GLP proteins were not hydroxylated, thus maintaining stability, resulting in H3K9 methylation with inhibitory effect on genes, and finally realizing the epigenetic regulation of FIH-G9a/GLP signaling axis on the invasion and metastasis of ovarian cancer [99]. FIH also has similar regulatory effects on HIF-1α and HIF-2α in physiological and pathological conditions [56]. Whether this indicates that G9a/GLP has direct effects on HIF-α activities via epigenetic regulation are still unknown. What we know is that G9a/GLP bind directly to HIF-1α protein in hypoxic tumors, both in vitro and in vivo, and catalyze monomethylation and dimethylation of HIF-1α at lysine 674, thereby inhibiting the transcriptional activity of HIF-1α and the expression of its downstream target genes [72]. For histone demethylases, the Jumonji domain (JMJD) containing protein and lysine-specific demethylase 1 (LSD1) involved in regulation of HIF stability [100]. The proteins of the Jumonji C (JmjC) containing family are mainly composed of 2-oxoglutarate (2OG)- and Fe^2+^-dependent histone demethylase, of which JMJD6 is an important member [101]. In recent years, JMJD6 has been thought to be related to the occurrence and development of a variety of tumors, including breast cancer, melanoma, oral cancer, glioblastoma, hepatocellular carcinoma, colon cancer, ovarian cancer and neuroglioma [101–110]. For instance, in ovarian cancer, Zheng et al. found that JMJD6 was highly expressed in tumor cells by tissue microarray immunohistochemical staining, and the high expression was associated with poor prognosis of patients [108]. According to the crystal structure characteristics of JmjC, they designed a JMJD6 inhibitor SKLB325, and tested the efficacy of the drug in vitro and in vivo, the results showed that the efficacy was good [108]. LSD1 is an flavin adenine dinucleotide (FAD)-dependent histone lysine demethylase, which can remove monomethyl or dimethyl from lysine 3 and lysine 9 of the histone 3 [100]. Abnormal LSD1 expression can be seen in a variety of cancers, such as blood, neuronal, thyroid, prostate, lung, colorectal, pancreatic, and breast cancers, suggesting that LSD1 can be developed as a molecular target for cancer [111, 112]. Instead of directly promoting the transcriptional activity of HIF, LSD1 has been reported to regulate the ubiquitin-degradation pathway of its protein, thereby affecting its activity [112]. In papillary thyroid carcinoma (PTC) tissues, high expression of LSD1 stabilizes HIF-1α to avoid its proteasomal degradation, and database prediction shows that HIF-1α is enriched near the miR-146a promoter region [112]. In vitro experiments, HIF-1α increased the expression level of miR-146a, and upregulated miR-146a inhibited the expression of target gene GABPA, finally leading to further malignant transformation of PTC [112]. The nude mouse model also further verified that LSD1 could up-regulate the expression of miR-146a [112]. LSD1 also has the function of non-histone lysine methylation [113], which is not discussed here. In addition, many recent studies have shown that LSD1 can be an important molecular target for the treatment of acute myeloid leukemia [114, 115], but therapies directly associated with HIFs activity need to be further explored.

### Histone acetylation and deacetylation are associated with HIF-α activity

p300/CBP and TAT-interactive protein 60 (TIP60) have acetylase activities that affect the transcriptional activity of HIF-1α, while HDAC4-6 in histone deacetylases (HDACs) regulate the stability of HIF-1α [2, 116, 117]. p300 and cyclic AMP response element-binding protein (CBP) are transcriptional coactivators with strong histone acetylase activity, which can regulate chromatin structure and make it more accessible to epigenetic regulators [118, 119]. p300 and CBP are tumor suppressor genes, and their mutations are involved in a variety of cancer pathways, affecting the development of tumors [118, 120, 121]. They bind to transcriptional activation regions of HIF-1α and HIF-2α genes, and acetylase activity of the p300/CBP complex, along with other deacetylases, is responsible for 70% target genes activation of the HIF pathway downstream [122]. Under nomorxia, FIH-mediated hydroxylation of an asparaginyl residue inhibited HIF-α recruitment of p300/CBP, thus affecting the further activation of the HIF pathway [123]. The acetylation of HIF-1α at lysine 709 by p300 increases the stability of HIF proteins, and SIRT1 deacetylates the p300 /CBP-associated factor (PCAF)-mediated lysine acetylation state of HIF-1α at lysine 674 to prevent p300/CBP recruitment and hypoxic-induced gene activation [124, 125]. And several studies have shown that inhibiting the functional activity of p300/CBP with inhibitors can be a potential target for tumor therapy [121, 123, 126]. TIP60 is a histone lysine acetylase that is involved in oncogenic pathways and affects the development and progression of tumors in a variety of ways [127]. TIP60 acts as a transcriptional coactivator of HIF-1α, affecting chromatin structure and regulating HIF target genes in colorectal cancer [128–130]. HIF-1α interacts with the component of the TIP60 complex, promoting TIP60 recruitment to chromatin (Fig. 1b) [128]. HIF-1α acts on TIP60, which leads to chromatin histone acetylation and then to the activation of polymerase II, which ultimately activates HIF-1α target genes transcription [128]. This suggests that TIP60 acts as a mediator, linking HIF-1α to HIF-1α target genes. In human cells, the 18 deacetylases are divided into four classes [131]. The class II is divided into two subclasses, IIa (HDAC4, HDAC5, HDAC7 and HDAC9) and IIb (HDAC6 and HDAC10), among which HDAC4, HDAC5 and HDAC6 are thought to be related to the regulation of HIF functional activity [2, 131, 132]. Within VHL-positive cancer cell lines, HDAC4 inhibition by shRNA increases HIF-1α protein acetylation levels, while HDAC4 overexpression decreases HIF-1α acetylation levels [133]. More, stable inhibition of HDAC4 in VHL-positive cells can not only reduce the transcriptional activity of HIF-1 and the expression of HIF-1 target genes, but also reduce the level of glycolysis [133]. Nucleus accumbens-associated protein-1 (NAC1), a member of the BTB/POZ gene family, can also interact with HDAC4 [134]. Intracellular accumulation of HDAC4 leads to reduced acetylation of HIF-1, but NAC1 binding to HDAC4 inhibits phosphorylation of HDAC4 at serine 246 and prevents nuclear export that leads to cytoplasmic degradation of HDAC [134]. In this context, the transcriptional activity and stability of HIF-1α was enhanced via NAC1-HDAC4-HIF-1α pathway [134]. HDAC5 has been found to promote transcriptional activation of HIF-1α and nuclear accumulation of HIF-1α [135]. The molecular chaperone Hsp70 acts as a substrate for HDAC5, and its deacetylation mediated by HDAC5 promotes the interaction of HIF-1 with Hsp90 [135]. HDAC6 plays an important role in hypoxic-induced reactions [136, 137]. HDAC6 is significantly down-regulated in liver cancer tissues, and low expression of HDAC6 is closely associated with poor prognosis [137]. HDAC6 promotes cell proliferation of hepatocellular carcinoma and HIF-1α and VEGFA expression, thereby promoting HIF-1-mediated angiogenesis in hypoxic conditions [137]. Multiple HDACs inhibitors can reverse the stabilization of HIF by HDACs, suggesting that HDACs, and specifically HDAC4-6, may be a molecular target for cancer therapy [138–141].

### Non-histone lysine acetylation is associated with HIF-α activity

HIF-α activity is also regulated by protein acetylation. For instance, lysine residue 532 (Lys-532) and lysine residue 709 (Lys-709) of HIF-1α protein can be acetylated in cancer cells [125, 142]. Arrest-defective protein 1 (ARD1) is functionally active as both N-terminal α-protein and ε-protein acetyltransferase activities in yeast, and was reported to be overexpressed in lung cancer, breast cancer, colorectal cancer and hepatocellular cancer [142, 143]. The lysine 532 of HIF is acetylated by ARD1, making HIF-1 more likely to interact with VHL for degradation [144]. Although ARD1 transcripts and proteins do decrease in hypoxic tumors, it still affects HIF-1α stability [142]. More, lysine 709 of HIF-1α is specifically acetylated by p300, enhancing HIF-1α stability and decreasing poly-ubiquitination within hypoxic tumors [125]. And HIF-1α K709A mutant protein were more stable and less p300 dependent than wild-type protein, suggesting that the interaction between HIF-1α and p300 is enhanced by HIF acetylation under hypoxia [125]. NAA10 usually has no acetylase activity [145]. However, it shows N-terminal acetylase activity similar to that of ARD1 when NAA10 is hydroxylated by FIH [142, 146]. In the aerobic condition, hydroxylated NAA10 acetylates HIF-1α protein and induces the protein destability [146], which suggesting that NAA10 mutation may contribute to tumor progression.

### Non-coding RNAs mediates epigenetic regulation on HIFs activity

Non-coding RNAs, mainly microRNA and lncRNA, play an important role in affecting HIF activity. Some lncRNAs have gradually become important regulators in the development of hypoxic tumors [147]. In nasopharyngeal carcinoma cells (NPC), both lncRNA PVT1 and lncRNA DANCR can interact with HIF-1α to influence NPC progression [148, 149]. Plasmacytoma variant translocation 1 (PVT1) is the first lncRNA gene discovered in Burkitt’s lymphoma, and its lncRNA that has been reported to play a role in promoting tumor progression [147]. PVT1 activates the acetyltransferase KAT2A, and then recruits TIF1β to promote the transcription of NF90 [148]. This improves the stability of HIF-1α protein and mRNA, and promotes further hypoxia-induced malignant phenotype of NPC [148]. Differentiation antagonizing non-protein coding RNA (DANCR) was identified as cancer-promoting gene in NPC cells, and was responsible for poor prognosis [149]. DANCR interacts with NF90/NF45 to stabilize HIF-1α mRNA and promote NPC development [149]. In breast cancer, miRNA-181c has an effect on the stability of HIF-1α [150]. Nuclear factor erythroid 2-like-2 (NRF2), an important regulator of genes related to oxygen pressure, increases miR-181c level in colon cancer cells [151]. Increased miRNA-181c led to a decrease in mitochondrial oxygen consumption rate and ATP production in cancer cells with NRF2 mutation, leading to HIF-1α destability [150]. It shows that down-regulation of HIF-1α mediated by miRNA-181c results in the inhibition of hypoxia-induced metabolic alterations within tumor cells with NRF2-silencing [150]. More, in pancreatic cancer, miRNA-646 and LncRNA-MTA2TR are also involved in the regulation of HIF-1α stability, respectively, thereby affecting HIF-1α accumulation in cancer cells [152, 153]. Migration and invasion inhibitory protein (MIIP) is identified as oncogenic blocker in pancreatic cancer [152]. miRNA-646 leads to reduced stability of MIIP mRNA and inhibition of MIIP gene expression [152, 154]. HIF-1α indirectly inhibits MIIP expression by activating miRNA-646 transcription, however, MIIP also has the ability to reduce the activity of histone deacetylase 6 (HDAC6) and thus promote HIF-1α acetylation and degradation [152]. It suggests that miRNA-646 indirectly induces HIF-1α stability via HIF-1α/miR-646/MIIP [152]. In addition, metastasis associated protein 2 (MTA2) is an metastasis-associated gene, and its lncRNA MTA2TR was overexpressed in gastric cancer and pancreatic cancer [153, 155]. MTA2 has nucleosome remodeling and histone deacetylase (NuDR) complex, and thus has the function of deacetylation [156]. lncRNA MTA2TR transcriptionally elevates MTA2 expression to increase the stability of HIF-1α protein through deacetylation [153].

The epigenetic regulation of these regulators on the functional activity of HIF-α involve the stability of HIF-α mRNA, the stability of HIF-α protein, and epigenetic reprogramming. Epigenetic factors that influence HIF-α stability are discussed here, but other factors that influence HIF-α activity, such as ATP-dependent chromatin remodelers, have been discussed in detail in other articles. Epigenetic modifications at the DNA, RNA and protein levels play an important role in HIFs availability, whether it’s methylation, acetylation or their reverse reactions, understanding the accurate mechanisms may help us establish drug reaction which will be discussed below, and exploring the pathological networks that haven’t found yet.

## Influence of intracellular metabolites on HIF-α stability through epigenetic regulation

The stability of HIF**-**α is the basis of its activity [116]. In the previous section, we discussed a number of epigenetic factors affecting HIF activity in hypoxic tumors. However, metabolites produced by hypoxic tumors during metabolism also play an important role in the stability of HIF**-**α [157]. Here, the epigenetic regulation of metabolites on the stability of HIF is discussed on the basis of genetic regulation.

### Effects of intracellular metabolites under genetic regulation on the stability of HIF-α

**Fig. 2:**
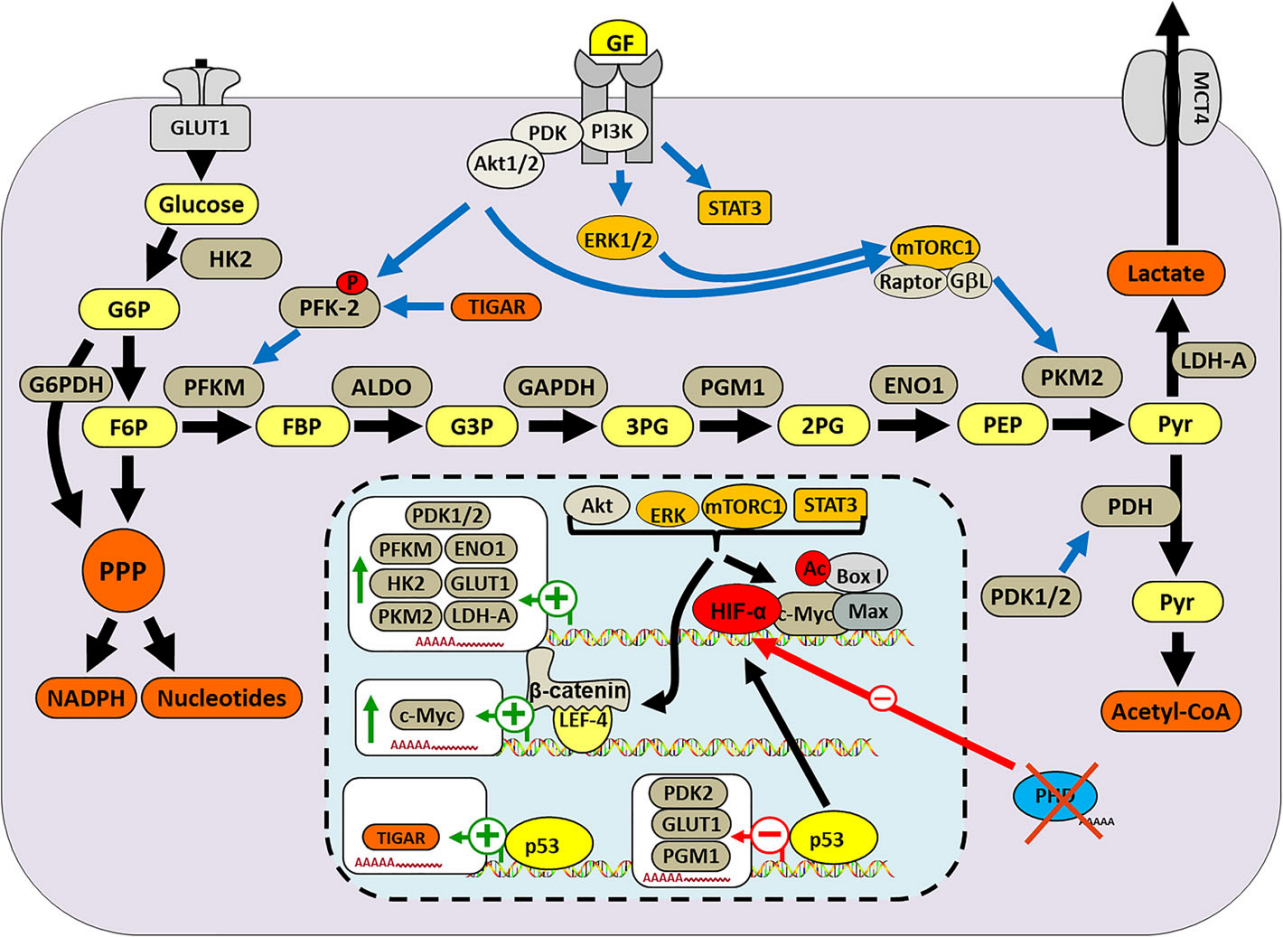
Impacts of intracellular metabolites on HIF-α stability through epigenetic regulation. In hypoxic tumors, rapid proliferation requires lots of intracellular metabolites to build macromolecules, including nucleotides and proteins. Up-regulated glycolysis sustains the demands of tumor cells for intracellular metabolites. HIF target genes encoding special enzymes are activated to produce various enzymatic proteins, such as PDK1/2, enolase 1 (ENO1), hexokinase 2 (HK2) and so on, which leading to elevated intracellular metabolites, in turn, these metabolites including succinate, fumarate, pyruvate, lactate and oxaloacetate etc. and associated pathways involved enzymes such as PI3K, PKB, promote HIF proteins stability with PHD loss-of-function. More, p53, β-catenin and so on, could also affect HIFs stability. PFKM: phosphofructokinase, muscle; GLUT1: glucose transporter 1; PKM2: pyruvate kinase isozymes M2; LDHA: lactate dehydrogenase A; ERK: extracellular signal-regulated kinases; PGM1: phosphoglucomutase-1; G6PDH: glucose-6-phosphate dehydrogenase; Aldo: Aldosterone; MCT4: monocarboxylate transporter 4; F6P: fructose 6-phosphate; FBP: fructose-1,6-bisphosphate; G3P: glycerol-3-phosphate; 3PG: 3-phosphoglyceric acid; 2PG: 3-phosphoglyceric acid; PEP: phospho enol pyruvate

In the past few years, numerous studies have shown that metabolism is passive, subordinate to the metabolic needs of the tumor, driven by the activation of oncogenes and the inactivation of tumor suppressor factors [158]. HIF-driven tumor metabolic remodeling activates multiple metabolic pathways, including pyruvate dehydrogenase kinase 1 (PDK1), BHLH Transcription Factor (MYC), pyruvate kinase M2 (PKM2), tumor protein P53 (TP53) [159, 160]. Many HIF target genes encode special enzymes, in turn, metabolites, including succinate, fumarate, pyruvate, lactate and oxaloacetate etc. affect HIF proteins stability due to loss-of-function of PHD (Fig. 2) [161–163]. For example, three succinate dehydrogenase (SDHB, SDHC and SDHD) and fumarate hydratase are reported to response to hypoxia, and aberrant function of these enzymes inhibit the process of mitochondrial respiratory [164]. Their mutation lead to increased ROS, and altered intracellular metabolites of TCA cycle as messengers to induce HIF stability [165]. In detail, inhibition of SDH genes coding aberrant enzymes lead to the loss-of-function PHDs with increased amount of succinate. As we discussed above, PHDs are responsible for the ubiquitylation leading to degradation of HIF-α, therefore the inhibition of PHDs leads to the accumulation PHDs substrates, above all HIF-α subunits [164]. Interestingly, among the accumulated substrates, succinate and fumarate could also contribute to HIF stability [165]. The recent study confirms this opinion that the FIH in concert with PHD/VHL in rapidly response to hypoxia, in turn, altered metabolites lead to HIF stability [56]. And authentic study speculated that lipopolysaccharide produced by gram-negative bacteria potently enhance the TCA-cycle intermediate metabolites succinate, performing a role of stabilizing HIF-1α accompanied with increased interleukin-1β, which finally mediating inflammation [161]. Isocitrate dehydrogenase isoform-1 (IDH1) and 2 (IDH2) mutations are considered to be associated with HIF-α stability in solid tumors, notably glioblastoma and acute myeloid leukemia (AML) [166, 167]. Mutant IDH proteins obtain neomorphic enzymatic activity that catalyze the transformation of α-ketoglutarate (α-KG) to R-2-hydroxyglutarate (R-2HG), which activates prolyl hydroxylase domain-2 that further leads to the degradation of HIF-α [168]. Moreover, non-catalytic enzymes associated with metabolic plasticity could also interact with HIF-α, such as fructose-1,6-bisphosphatase (FBP1) and PKM2. In ccRCC, the loss of FBP1 has been identified, and it may function as repressor of HIF-α via binding to the degradation domain of HIF in the nucleus, and further inhibit ccRCC progression. In human pancreatic adenocarcinoma, high level PKM2 expression interacts with NF-κB and HIF-1α to induce the activation of HIF target gene VEGF. In contrast to FBP1, PKM2 could function as a coactivator to directly bind with HIF-1α by facilitating the recruitment of p300 [16, 37, 169].

### The epigenetic regulation of intratumoral metabolites on stability of HIF-α

**Fig. 3:**
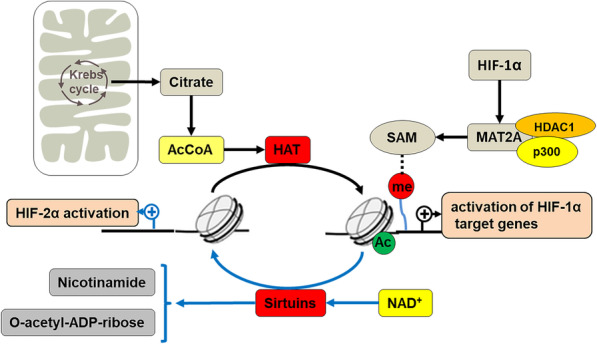
The epigenetic regulation of acetyl-CoA, NAD^+^ and SAM on stability of HIF-α. In the tricarboxylic acid cycle of mitochondria, AMPK phosphorylates acetyl-CoA carboxylase (ACC), leading to increased acetyl-CoA in tumor cells. The activity of histone acetylases (HATs) such as p300/CBP, which uses acetyl-CoA as its substrate, is increased accordingly. The acetyl-CoA leads to increased p300/CBP activity, so recruitment of p300/CBP by HIF-1α results in substantial activation of HIF-1 downstream genes. In hypoxic tumors, residual Sirtuins catalyze the deacetylation of histones in NAD^+^ − dependent reactions to produce a deacetylated substrate, o-acetyl ADP-ribose, and niacinamide. This results in changes in HIF-α and its downstream gene activation. HIF-1α recruits p300 and HDAC1 to the MAT2A promoter, leading to high expression of MAT2A. Up-regulation of MAT2A regulates genomic DNA methylation status by affecting SAM levels

During tumor progression, tumor metabolism is regulated by a number of metabolic regulators, including HIF-1α, AMPK, mTOR, and PPAR gamma coactivator 1 alpha (PGC-1α), wherein AMPK indirectly regulates the stability of HIF-1α through metabolites [170, 171]. AMPK is a heterotrimeric Ser/Thr kinase complex that acts as a cellular sensor for energy state and ROS in tumor cells to maintain intracellular energy homeostasis [170]. Acetyl-CoA is a key metabolite that links metabolism to transcription and chromatin structure [172]. AMPK regulates the levels of acetyl-CoA and NAD^+^ to regulate the activities of histone acetylases (HATs) and HDACs, thus affecting the activity of HIF-1α (Fig. 3) [173]. AMPK phosphorylates and inhibits acetyl-CoA carboxylase (ACC), thereby increasing the level of acetyl-coA in tumor cells [174]. And since acetyl-CoA is the substrate for all KATs, lysine acetyltransferase activity is increased [175]. For example, the p300/CBP family is a type of KATs whose activity can be enhanced by acetyl-CoA, which further promotes its binding to HIF-1α and activating most of the downstream target genes of HIF-1α [121]. HDAC can be divided into four classes, among which, class I, class II, class IV are Zn^2+^-dependent aminohydrolases, while class III use NAD^+^ as a cosubstrate, thereby exerting the role of deacetylase to affect the stability of HIF-1α and HIF-2α and thus regulate HIF-driven metabolic activities [176]. The specific mechanism by which Class III, also known as Sirtuin family, acts on HIF-α is discussed in the next section. In normal cells, pyruvate, as the end product of glycolysis, is decomposed by pyruvate dehydrogenase (PDH) into acetyl-CoA and CO_2_ in mitochondria, while generating NADH. However, in hypoxic tumor cells, HIF-1 target gene PDK1 is activated and PKD1 inactivates PDH, resulting in pyruvate being excluded from mitochondria [27]. Finally, the levels of acetyl-CoA and NAD^+^ in hypoxic tumors were reduced, which seemed to contradict their ability to stabilize HIF-1α and HIF-2α and promote tumor progression [177, 178]. However, pyruvate fails to enter the electron transport chain, which reduces oxidative phosphorylation levels and ROS production [178, 179]. ROS can promote death of tumor cells through many pathways, such as ferroptosis [46, 180, 181]. Therefore, compared with cell death caused by ROS, tumors prefer to choose other conventional metabolites, such as succinate, glutamate and fumarate, to maintain tumor progression [157].

The production of SAM also plays an important role in DNA methylation in tumor metabolic fitness and plasticity [27, 182]. SAM can be synthesized from methionine and ATP catalyzed by methionine adenosyltransferases (MATs), and is involved in the regulation of genomic DNA methylation status [75, 183]. For instance, SAM metabolism affects DNA methylation status and lead to metabolic reprogramming in liver cancer progression and prognosis [184]. Down-regulation of the liver-specific MAT1A gene encoding isozymes MATI/III and up-regulation of the MAT2A gene encoding isozyme MATII occur in hepatocellular carcinoma, and the resulting MAT1A: MAT2A switch leads to a decrease in SAM level [184, 185]. However, studies showed that the MAT1A: MAT2A switch and low SAM level are associated with CpG sites methylation of MAT1A and MAT2A promoters in HCC [186]. With the involvement of HIF-1α, SAM affects the activity of ERK1/2 by interfering with DUSP1 [187]. As a member of the AMPK family, active ERK1/2 phosphorylates serine residue of DUSP1, leading to DUSP1 ubiquitylation and degradation [187]. However, SAM treatment stabilizes USP1 at both mRNA and protein levels, suggesting that AMPK is regulated by SAM [188]. Interestingly, HIF-1α stabilizes the ERK1/2 target gene FOXM1 and mediates metabolic reprogramming regulated by AMPK under hypoxia in HCC [189]. Hypoxia decreases SAM levels in HCC cells, leading to reduced genomic DNA methylation levels [190]. Mechanistically, hypoxia-induced HIF-1α recruits p300 and HDAC1 to the promoter of MAT2A, and the resulting up-regulation of MAT2A plays an important role in decreasing SAM level with specific mechanism (Fig. 3) [190]. In addition, reliable studies have shown that MAT2B, an important regulator of MAT2A, inhibits the activity of MAT2A at high SAM levels, and acts as a co-activator of MAT2A at low SAM levels [191]. These results suggest that high expression of MAT2A, which is activated by HIF-1α in hypoxia, can be negatively regulated by MAT2B, resulting in the decrease of SAM instead [191]. This further suggests that in hypoxic tumors, SAM biosynthesis may affect HIF-1α activity by regulating genomic DNA methylation status.

In general, there are many factors affecting the stability and activity of HIF-α, and metabolites are one of the most important aspects, which is why we discuss it separately. In terms of genetic regulation, the effect of intracellular metabolism on HIF-α is usually involved in metabolic reprogramming, especially glucose metabolism, but evidence on the epigenetic regulation of HIF-α activity by metabolites is limited.

## Role of HIF-α in nutrient deprivation during cancer progression through epigenetic regulation

The hypoxic tumors are characterized by oxygen deficiency and nutrient deprivation, which mainly involves glucose and amino acids [192, 193]. Growing evidence have shown that tumors prefer glycolysis, even in aerobic conditions [14]. Histone deacetylases and non-coding RNAs both play important roles in HIF-involved glucose depletion [194, 195]. In addition, fatty acid metabolism also constitute an important part of tumor metabolic reprogramming [196]. Here, we briefly reviewed not only HIF-mediated genetic regulation of tumor metabolism, but also epigenetic regulation of glucose deprivation. Here, we briefly review the overall glucose metabolism of tumors by genetic regulation, and try to discuss epigenetic regulation of glucose metabolism.

### Metabolic reprogramming with canonical genetic regulation

The patterns of tumor metabolism are very different from those of surrounding tissue [14]. Only 10% of ATP in normal tissues comes from glycolysis, and the rest 90% comes from metabolic activities in mitochondria [157]. However, 50% of ATP needed by tumors comes from glycolysis and mitochondria, respectively [157]. HIF-dependent glycolysis is a linear metabolite processing process involving the expression of several genes, including glucose transporter (GLUT) genes, enzyme genes that break down glucose into pyruvate, and enzyme genes that clear pyruvate [197]. HIF-1 increases the rate of glucose internalization by activating the expression of GLUT1 and GLUT3 [198, 199]. Increased GLUT1 and GLUT3, induced by HIF-1, transport glucose from high concentration to low concentration into tumor cells, and glucose entering the cells can be used for multiple purposes, including glycogen synthesis, protein modification, and pentose shunt [157]. In the cytoplasm, hexokinase (HK) converts glucose to glucose 6-phosphate (G6P), which is then converted to glucose 1-phosphate (G1P) by phosphoglucomutase 1 (PGM1) [200]. G1P can be further transformed into UDP-glucose to form the constituent unit of glycogen. When glycogen is decomposed in the cytoplasm, G1P can be regenerated due to the functions of glycogen phosphorylase (PYG) and a debranching enzyme [200]. And G1P can be converted by PGM1 into G6P to participate in glycolysis [200]. Studies have shown that epithelial cells store energy in the form of glycogen, but glycogen is not a major source of energy for tumors [201]. In addition to glycogen metabolism, the main fate of glucose is the synthesis of pyruvates under the activities of the 6-phosphofructo-2-kinase/fructose-2,6-bisphosphatase (PFKFB) enzymes induced by HIF-1 [157]. That is, glucose, 2 ADP and 2 NAD^+^ are converted into 2 pyruvates, 2 ATP and 2 NADH during glycolysis [157]. In most hypoxic tumors, pyruvate, the end product of glycolysis, is the primary carbon source, and HIF-1-induced pyruvate dehydrogenase kinase 1 (PDK1) is a protein kinase that phosphorylates and inactivates the mitochondrial enzyme pyruvate dehydrogenase (PDH) responsible for pyruvate catalysis [202]. PDH catalyzes pyruvate into acetyl-CoA and CO_2_ in mitochondria to produce NADH, but with the inactivation of PDH, this process is blocked [203, 204].

**Fig. 4:**
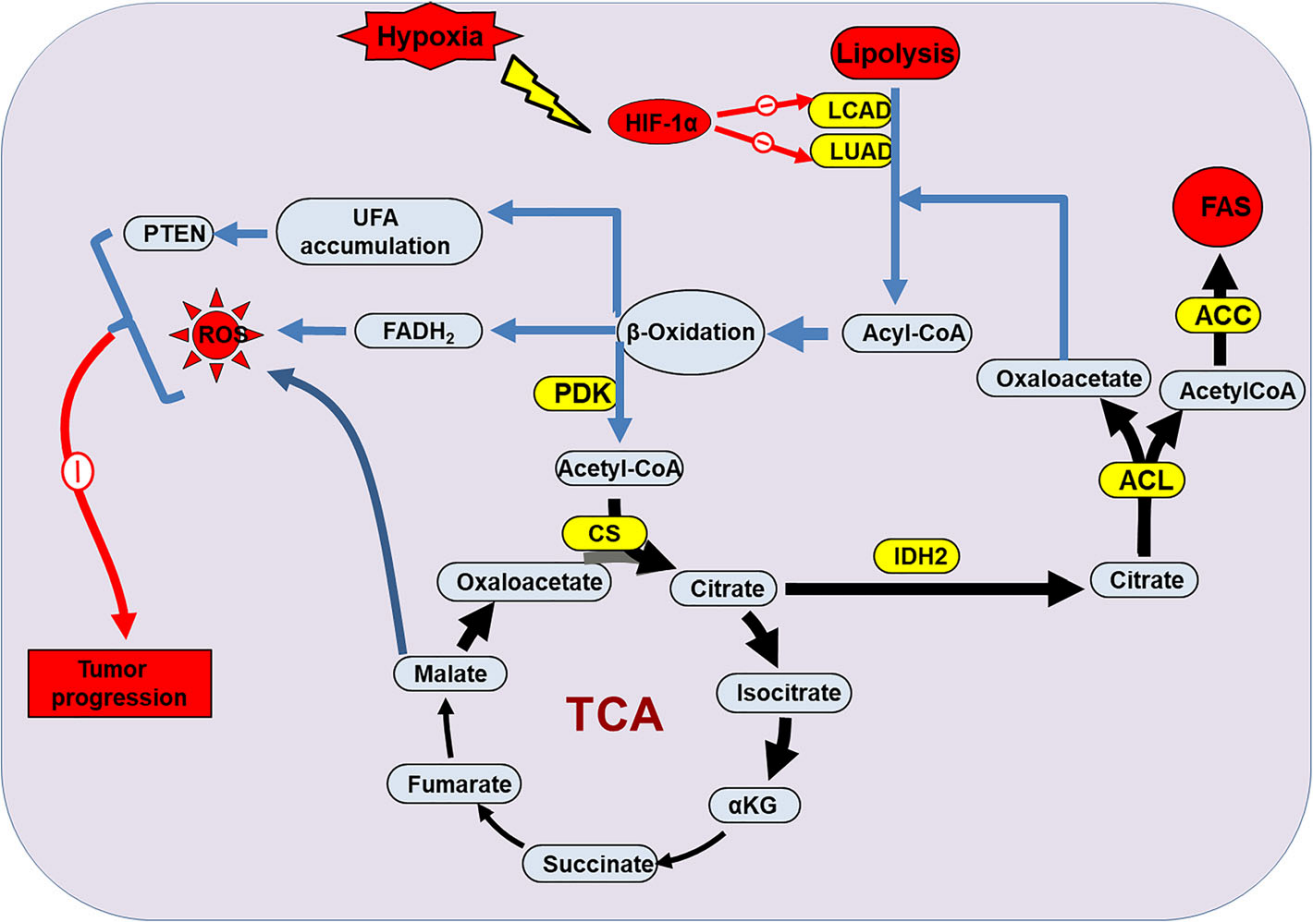
Role of HIF-α on fatty acids metabolism during cancer progression via epigenetic regulation. Two FAO enzymes, the acyl-CoA dehydrogenases MCAD and LCAD, are inhibited by the activated HIF-1α under hipoxia. LCAD and MCAD loss disturbs the process of FAO which leads to ROS alteration via TCA (tricarboxylic acid) cycle and separate LCAD loss inhibits PTEN pathway, which finally mediate resistance to tumor progression. HIF-1α loss-of-function may rescues the resistance and contributes to cancer progression. CS: citrate synthase; ACL: acetone-cyanohydrin lyase; ACC: acetyl-CoA carboxylase alpha

Fatty acid metabolism also exist in tumor cells, promoting tumor progression. It is important for tumors to reverse or mitigate the adverse effects of nutrient deprivation because the cells at the core site are highly plastic and contribute to tumor progression [27, 205]. Thus, HIF activates a series of enzymatic genes that perform metabolic reprogramming through the fatty acid (FA) synthase system [165, 206, 207]. For example, the transcriptional activity of genes coding fatty acid synthases, particularly fatty acid synthase (FASN) and acetyl-CoA carboxylase (ACC), leas to elevated de novo FA synthesis [208]. HIF-1α not only inhibits pyruvate metabolic pathway in mitochondria during glycolysis, but also inhibits fatty acid oxidation (FAO) to promote tumor progression (Fig. 4) [209–211]. Mechanistically, two FAO enzymes, medium-chain acyl-CoA dehydrogenase (MCAD) and long-chain acyl-CoA dehydrogenase (LCAD), reduce ROS levels to promote tumor cell proliferation, and loss-of-function of LCAD further accelerates cancer progression via involving in phosphatase and tensin homolog (PTEN) pathway [209]. In addition, HIF-2α has been found to be highly expressed in patients with liver disease, and with the activation of its target genes, such as SRT1 and AMPK, liver fibrosis is intensified, suggesting that HIF-2 inactivation can reverse the progression of liver cancer [212, 213]. In the case of tumor nutritional deprivation, fatty acid metabolism regulated by HIF-1α and HIF-2α have gradually shown its importance, but more work, such as epigenetic regulation, still needs to be further studied.

### The histone deacetylases regulate glucose metabolism in tumors via epigenetic regulation

Limited literature suggests that histone deacetylases, especially members of the Sirtuin (SIRT) family, play an important epigenetic role in HIF-regulated glucose metabolism [195, 214]. SIRT family can be divided into four classes, SIRT1, SIRT2 and SIRT3 belong to class I, SIRT4 belong to class II, SIRT5 belong to class III, and SIRT6 and SIRT7 belong to class IV [215]. Among them, SIRT1, SIRT3, SIRT6 can play regulatory roles in multiple links of glucose metabolism regulated by HIFs [195, 216]. Under nomorxia, SIRT1 promotes HIF-1α degradation by stabilizing intracellular VHL transcripts, and SIRT1 can further deacetylate HIF-1α in the nucleus, affecting the interaction between HIF-1α and p300 to affect the activation of a range of metabolism-related target genes [124]. In hypoxic tumors, the activity of SIRT1 is inhibited by the decreasing NAD^+^, but residual SIRT1 deacetylates HIF-2α and activate HIF-2α [217]. Therefore, it suggests that SIRT1 may form a dynamically balanced pool in hypoxic tumor cells, regulating the activities of HIF-1α and HIF-2α according to the oxygen availability [195, 218]. In tumor cells, SIRT3, as the major mitochondrial deacetylase in the Sirtuin family, promotes mitochondrial respiration and inhibits HIF-regulated glycolysis [219]. SIRT3 can deacetylate and activate various mitochondrial metabolic enzymes involved in TCA and FAO enzymes, including LCAD, 3-hydroxy-3-methylglutaryl CoA synthase 2 (HMGCS2), isocitrate dehydrogenase 2 (IDH2) and glutamate dehydrogenase (GDH) to block the supply of glycolysis to the tumor cells [219]. SIRT6-defiencicy mice showed fatal hypoglycemia, suggesting that SIRT6 is involved in glucose metabolism [220]. SIRT6, an H3K9 deacetylase, inhibits transcriptional activation of HIF-1α in non-tumor cells and reduces the level of glucose metabolism, but SIRT6 knockdown increased glucose uptake in mice, inhibited mitochondrial respiration, and effectively responded to nutrient deprivation [220]. However, in tumor cells such as colon cancer cells, SIRT6 binds directly to the hypoxia-responsive elements of the glycometabolism genes and blocks HIF-1α-induced glycometabolism without activating oncogenic pathway [219]. This suggests that SIRT6 acts as a tumor suppressor in the development of tumors by inhibiting glucose metabolism [219]. Other members of the Sirtuin family, such as SIRT7, have also been shown to influence HIF-induced metabolic activity via decreasing HIF-1α and HIF-2α, but the specific mechanism remains to be investigated [221].

### The role of non-coding RNAs in glucose metabolism via epigenetic regulation

There is considerable evidence that non-coding RNAs, including microRNAs and lncRNAs, regulates HIF-induced metabolic activity [194, 222]. In liver cancer, miR-3662 can inhibit the growth and proliferation of liver cancer cells by negatively regulating HIF-1α-mediated glycolysis [223]. miR-3662 is low expressed in liver cancer tissues, and this low expression is closely related to tumor size, multiplicity and metastasis [223]. Mechanistically, miR-3662 down-regulates HIF-1α expression, thereby reducing lactate level, glucose metabolism rate, intracellular glucose-6-phosphate content, ATP level, but increasing oxygen consumption rate, suggesting that miR-3662/HIF-1α axis is closely related to glucose metabolism reprogramming [223]. In colorectal cancer cells, miR-23a, miR-27a and miR-24 were found to be upregulated microRNAs that collectively regulate the glucose metabolic network [224]. HIF-1α binds to the promoter of miR-23a ~ 27a ~ 24 cluster formed by miR-23a, miR-27a and miR-24, promoting the expression of miRNA clusters in HCC cells [224]. miR-24/VHL/HIF-1α forms a double-negative feedback regulatory pathway that can enhance the effect of HIF-1α and miR-23a ~ 27a ~ 24 cluster, greatly regulating the metabolic network of colorectal cancer and shifting the metabolic balance of normal cells to glycolysis [224]. Homeobox A9 (HOXA9), as the target gene of miR-365, can inhibit glycolysis by regulating HIF-1α and the downstream glycolysis genes [225]. However, HOXA9 is down-regulated in cutaneous squamous cell carcinoma (cSCC), which means that the miR-365-HOXA9-HIF-1α axis promotes glycolysis [225]. In chronic myelogenous leukemia (CML), curcumin was found to up-regulate the expression of miR-22 and down-regulate its target gene importin 7 (IPO7), thus affecting the nuclear transport of HIF-1α [226]. Thus, the target genes of HIF-1α related to glycolysis were down-regulated [226]. In acute myeloid leukemia (AML), the up-regulated expression of LncRNA urothelial carcinoma-associated 1 (LncRNA-UCA1) inhibited the adriamycin (ADR)-based chemotherapy effect by negatively regulating glycolysis [227]. LncRNA-UCA1 can be directly bound to miR-125a as ceRNA, and in HL60andHL60/ADR cells, target gene hexokinase 2 (HK2) of miR-125a can be positively regulated by lncRNA-UCA1 [227]. Importantly, lncRNA-UCA1 overexpression can reverse the HIF-1α-dependent glycolysis inhibition mediated by miR-125a in HL60andHL60/ADR cells, showing that lncRNA-UCA1 could inhibit glycolysis through miR-125a/HK2 pathway and plays an active role in overcoming chemotherapeutic resistance to AML in children [227].

Nutritional deprivation is one of the most common metabolic phenomena in hypoxic tumors, and with the involvement of the HIF family, tumors use a variety of metabolic pathways to shape the plasticity of tumor cells and their microenvironment in order to cope with or reverse undesirable situations. In terms of phenotypic plasticity, in addition to the glucose and fatty acid metabolism mentioned above, there are also amino acid metabolism that have not been discussed. However, in terms of the epigenetic regulation of HIF-related nutrient deprivation, the reference basis is still limited, mainly focusing on histone deacetylases SIRT family and non-coding RNAs. Among them, some epigenetic regulations are concentrated on HIF target genes, suggesting that seeking more downstream HIF genes may be a way to deepen our understanding of epigenetic regulation in hypoxic tumors.

## Role of HIF-α in tumor microenvironment through epigenetic regulation

Many studies have shown that genetic changes fail to fully explain tumor progression, invasion and metastasis, and some epigenetic changes occurring in tumor cells have profound impacts on tumor progression [228]. Both microenvironment cues and intracellular alterations promote epigenetic regulation [228–230]. Here we briefly summarize the HIF-α**-**related epigenetic regulation in the tumor microenvironment between tumor cells and myeloid cells.

The tumor microenvironment mainly includes inflammatory cells, accessory fibroblasts and extracellular matrix (ECM) components, which have high phenotypic plasticity [231]. Cell-to-cell contact, secretion of soluble factors and release of exosomes can lead to tumor microenvironmental disorders that have important effects on both genetic and epigenetic characteristics [232–235]. Infiltrating and tissue-resident myeloid cells are important regulators of innate and adaptive immunity [236]. During inflammation, these cells can adapt to microenvironmental conditions and acquire specific functions, including phagocytosis and the production of pro-inflammatory cytokines [237]. This myeloid plasticity is driven in part by epigenetic regulation that maintains a stable phenotype after activation, such as demethylation by TET family member TET2 enzyme [237, 238]. There is increasing evidence that pathological activation and differentiation of myeloid cells is a marker of cancer [238]. TET2 is mutated in some malignant myelopathy, indicating its important role in the proliferation and differentiation of myeloid cells [239]. Hydroxylation of 5-methylcytosine (5meC) was inhibited in myeloid cancer cells with TET2 mutations, which may be related to the altered methylation levels observed in these cells [240, 241]. Activation and differentiation of tolerogenic myeloid cells, including myeloid-derived suppressor cells (MDSCs), regulatory dendritic cells (regDCs) and tumor-associated macrophages (TAMs), are regulated not only by gene expression, but also by epigenetic regulation in tumor environments [235]. TET2 has been found to inhibit the expression of inflammatory cytokines IL6, which has resolved the inflammation of innate myeloid cells [242]. Interestingly, the inhibition of IL6 by TET2 requires recruitment of HDAC2 [242]. In addition, HIF-1α deletion in glioblastoma cells was shown to increase TET2 transcription and translation levels and further promote ascorba-induced and TET2-dependent 5hmC, suggesting that HIF-1α is involved in regulating TET2 expression and 5hmC levels in malignant cells [243]. Thus, HIF-1α regulation of TET2 in human metastatic melanoma can also be reflected in myeloid malignant cells. The pathological switch of immature bone marrow cells to tolerogenic MDSCs is driven by tumor-derived molecules as well as epigenetic regulation [244]. MDSCs are heterogeneous populations composed of pathologically activated immature myeloid cells, which inhibit anti-tumor immune response and promote tumor angiogenesis and tumor invasion [245, 246]. In ovarian cancer, MDSCs play an inhibitory role through prostaglandin E2 (PGE2), which not only enables normal dendritic cells (DCs) to differentiate into tolerogenic MDSCs, but also mediates MDSCs-derived PGE2 to directly inhibit CD8^+^ T cell function [235, 247]. Moreover, the generation of PGE2-mediated monocytic MDSCs is dependent on the up-regulation of DNA methyltransferase 3A (DNMT3A) [248]. In MDSCs, the upregulation of DNMT3A in is accompanied by specific DNA methylation and immunogenic-related gene suppression, while the downregulation of DNMT3A would lead to the decreased hypermethylation level and the loss of immunosuppressive activity of MDSCs [248]. Interestingly, primary MDSCs isolated from ovarian cancer patients showed similar hypermethylation characteristics associated with PGE2-dependent DNMT3A overexpression [248]. However, DNMT3A was found to methylate and inactivate the HIF-2α gene EPAS1, and inactivation of DNMT3A in the early stages of tumor cell progression leads to abnormal activation of EPAS1 [249]. This allows cancer cells to take advantage of the HIF-2α pathway in the hypoxic tumor microenvironment to form a cell mass larger than the oxygen diffusion limit [249].

As an important component of the microenvironment, stromal cells also have close epigenetic links with tumor cells [250, 251]. For example, histone methyltransferase enhancer of zeste homolog 2 (EZH2) regulates its target gene HIF-1α to influence the status of the tumor microenvironment [252]. The specific mechanism will be discussed in other sections. However, the involvement of HIF-α in the epigenetic mechanisms between immune cells and tumor cells remain unclear. The reason for this phenomenon may be that the tumor microenvironment is a complex system, and a lot of epigenetic regulation in tumor progression will affect the microenvironment and tumor cells plasticity, so HIF-α may not be the only participant in such a complex epigenetic regulatory network.

## Role of HIF-α on extracellular matrix remodeling through epigenetic regulation

The extracellular matrix (ECM) is a dynamic, non-cellular and three-dimensional structure that occurs in all tissues [253]. ECM is composed of collagen, elastin fibrils, proteoglycan, glycosaminoglycan, glycoprotein and protease, which are interconnected to form a dynamic cell regulatory niche and provide structural stability [254]. Studies have shown that ECM is sustainably remodeled to play an important role in the proliferation, migration, adhesion, invasion and metastasis of tumor cells [255, 256]. In intratumoral hypoxia, HIF-1α activates genes encoding collagen prolyl hydroxylases, such as P4HA1 and P4HA2, and collagen prolyl hydroxylases, such as PLOD2 [257]. In this context, epigenetic regulation mainly involves non-coding RNAs [258].

VEGF is an important factor in the regulation of tumor angiogenesis [259]. Hypoxia is a major regulator of VEGF expression via HIFs [260]. HIFs and its target genes such as epidermal growth factor and platelet-derived growth factor coordinate VEGF expression within tumor cells [260, 261]. Studies have shown that miRNAs targeting the HIF-VEGF axis may have important effects on angiogenesis [258, 262]. For example, miR-484 works by targeting VEGFB and VEGFR2 pathways to determine chemotherapy resistance in serous ovarian cancer [262]. In addition, miR-120 is considered to be the most important miRNA induced in epithelial cells [263]. miR-210 is overexpressed in normoxic epithelial cells, which stimulates the formation of capillary-like structures and promotes VEGF-driven cell migration [264]. However, hypoxia-induced mir-210 blockade could inhibit the above phenomena, because during HIF-induced hypoxia, mir-210 directly down-regulated its target gene tyrosine kinase ligand Ephrin-A3 to eventually inhibit the survival, migration and differentiation of the endothelial cells [264]. In this case, VEGF-mediated angiogenesis is blocked in hypoxia, which may be further hypothesized to be the same in epithelial tumors [265]. ECM plays a fundamental role in controlling angiogenesis by providing basic structural support for cytokines, direct signal transduction function, and scaffolding [259, 266]. Thus, ECM has long been considered essential for all stages of angiogenesis [267]. However, a recent study has shown that VEGF also plays an important role in ECM remodeling in metastatic colorectal cancer (mCRC) [268]. In patients with mCRC who were treated with bevacizumab prior to surgical intervention, deposits of hyaluronic acid (HA) within the tumor were found [268]. At the same time, anti-VEGF therapy was found in the homologous mCRC mouse model to significantly increase the expression of HA and sulfated glycosaminoglycans (sGAGs), but not significantly change collagen deposition, indicating that tumor hypoxia induced by treatment promotes ECM remodeling [268]. However, we do not know exactly which HIF-α is involved in regulating hypoxic activity in this study, and further investigation is needed.

There are many factors that regulate ECM remodeling, but miRNA is undoubtedly an important link. The regulation of miRNA on ECM remodeling mainly focuses on VEGF. Whether other enzyme genes involved in ECM remodeling also generally accept the regulation of miRNA or even other non-coding RNAs has not yet formed an understanding paradigm.

## Role of HIF-α in metastasis through epigenetic regulation

Tumor metastasis is a major challenge in clinical management and is often associated with high mortality from cancer because it cannot be cured with conventional chemotherapy and radiotherapy [269]. Metastasis is a complex dynamic process in which highly aggressive tumor cells acquire the ability to spread from the primary site to new tissues and organs and eventually survive at distant sites [270]. Hypoxia has become a key microenvironmental factor regulating metastasis, especially HIF signaling pathway [271]. From a genetic point of view, metastasis may result from a succession of genetic mutations [272]. Therefore, for a long time, the use of sequencing technology to analyze the mutation profiles on a genome-wide scale has been a hot topic [272]. However, in recent years, it has been suggested that traditional mutation drivers cannot explain the phenomena observed in experiments or clinical practice, so it has been suggested that epigenetic regulation may play an extremely important role, especially in hypoxia-linked metastasis [271].

Activation of HIF signaling regulates multiple stages in the cascade of metastasis, including invasion and migration, intravasation and extravasation, establishment of the pre-metastatic niche, and survival and growth at distant organ sites [269]. Epigenetic regulation of gene expression profiles usually affects metastasis in three ways: (1) regulation of key genes involved in metastasis; (2) extensive epigenetic remodeling due to changes in cell state; and (3) epigenetic regulation of non-coding RNA in tumor metastasis [272–276]. In the first way, epigenetic regulators of HIF are JMJD2C, TET1 and VHL [99, 277–279]. JMJD2C specifically interacts with HIF-1α, and HIF-1α can recruit JMJD2C into the hypoxia response elements of HIF-1 target genes [277]. JMJD2C reduces the level of trimethylation on the histone H3 at lysine 9 (H3K9), and activate the genes lysyl oxidase-like protein 2 (LOXL2) and L1 cell adhesion molecule (L1CAM) that can promote lung cancer metastasis [277]. TET1, as DNA demethylase, is thought to be related to tumor metastasis [280]. TET1, as DNA demethylase, is thought to be associated with tumor metastasis, and hypoxia increases the expression of TET1 in a HIF-1α-dependent manner [281, 282]. Because the binding of HIF-1α to HRE of target genes depends on the methylation level of CpG, TET1 can regulate the HIF-1α target genes by regulating the methylation state of HRE [278]. Moreover, TET1 E2082K mutant inhibits the TET1-enhanced cell migration in colon cancer [278]. VHL regulates hypoxic signaling by controlling the ubiquitination and degradation of HIF-α protein, thus genetic or epigenetic inactivation of VHL leads to constitutive activation of HIF-1α and HIF-2α [283]. Epigenetic modification of prometastatic genes in VHL-deficient ccRCCs cell subsets was found to increase the expression of metastasis-related HIF target genes [284]. For example, studies on two important pro-metastatic genes chemokine (C-X-C motif) receptor 4 (CXCR4) and cytohesin 1 interacting protein (CYTIP) have found that ablation of the polycomb repressive complex 2 (PRC2)-dependent histone H3 lysine 27 trimethylation activates the expression of HIF-driven CXCR4 [283]. To promote chemotactic cell invasion, DNA methylation is lost, leading to HIFF-driven CYTIP gene expression to protect tumor cells from death chemical signals [283]. In the second way, epithelial-mesenchymal transition is a beautiful example [274]. EMT is a key process in tumor metastasis, that is, the transformation of well-differentiated epithelial cells into less differentiated mesenchymal cells makes it easier for tumors to invade adjacent tissues and spread to distant organs for survive [274, 285]. EMT induced by hypoxic signaling can be regulated by HDAC3 and WD repeat containing protein 5 (WDR5) to regulate the inhibition of epithelial genes and the activation of mesenchymal genes [286]. In hypoxia, HDAC3 and WDR5 are activated by HIF-1α to recruit histone methyltransferase (HMT) complexes to increase histone H3 lysine 4-specific HMT activity and promote the expression of mesenchymal genes [286]. In addition, HDAC3 also acts as a cofactor to inhibit epithelial gene expression, and WDR5 knockout can eliminate mesenchymal gene activation [286]. This suggests that HDAC and WDR5 can jointly epigenetically regulate the metastatic phenotype of cancer under the regulation of HIF-1α [286]. For the third way, both lncRNA and miRNA can interact with HIF respectively or jointly to regulate tumor metastasis [149, 287, 288]. For example, the taurine upregulated gene 1 (TUG1), as a potential oncogene, has been found to be abnormally expressed in osteosarcoma (OS) and has been associated with distant metastasis [288]. TUC1 expression was significantly increased in OS tissues [288]. TGF-β from cancer-associated fibroblasts (CAFs) can up-regulate the expression of TUG1, and the crosstalk between CAFs and OS activate TUG1 to generate lncRNA TUG1 and promote the metastasis of OS [288]. The specific expression of TUG1 competitively keep HIF-1α mRNA 3’UTR from miR-143-5p [288]. In addition, in hepatocellular carcinoma, lnc RNA ubiquitin conjugated enzyme E2C pseudogene 3 (UBE2CP3) has been reported as an oncogene that promotes tumor metastasis [289]. UBE2CP3, which is highly expressed in hepatocellular carcinoma tissues, promotes human umbilical vein endothelial cell (HUVEC) proliferation, migration, and tube formation through the ERK/HIF-1/p70S6K/VEGFA axis in vitro and in vivo compared to paracancer tissues, leading to upregulation of VEGFA expression [289]. Recent studies have shown that methylation leads to miRNA silencing and that these silenced miRNAs are reactivated by demethylation [275, 276, 290]. The re-expression of these silenced miRNA can regulate the tumor microenvironment and promote tumor metastasis [291]. This suggests that global hypomethylation is a feature of cancer cells, and that the crosstalk between HIF-α and miRNA may also involve methylation changes [291].

Tumor metastasis is often associated with poor prognosis and can be regulated by both HIF-α and epigenetics. Although disordered epigenetic patterns in cancer cells have long been identified, they have for a long time been ignored. But with the discovery of promoter hypermethylation leading to gene silencing and global hypomethylation in cancer cells, we have gained a deeper understanding of epigenetic disorders involved in HIF-α in cancer cells. However, many epigenetic mechanisms do not directly regulate HIF-α or are directly regulated by HIF-α, suggesting that these mechanisms are complex and that other intermedi are involved, which need to be further explored.

## Hypoxia and targeted therapy via epigenetic interference

Given the association between hypoxia and chemoresistance, radioresistance and targeted therapies, hypoxia has been regarded as a therapeutic target, and epigenetic therapies, particularly anti-angiogenic treatment, VHL-HIF axis therapy, bromodomain extralterminal proteins (BET) inhibition and other therapies to be argued etc., are of great interests. Clinically, resistance to multi-therapies has been observed, and the mechanisms behind poor outcomes vary with treatments. For example, resistance to radiotherapy is mediated by ROS which produced by ionizing radiation, however, hypoxic condition restrains the ROS production and further limits the damage to DNA. Also, chemicals can’t reach the tumors in sufficient quantities due to abnormal vascularization and up-regulation of HIF signaling etc., which leads to chemoresistance [292].

### Anti-angiogenic therapy

Anti-vascular therapy has long played a pivotal role in oncotherapy. Angiogenesis is a hallmark of solid tumors, and the process of which is governed by VEGF over-expression induced by hypoxia [293, 294]. VEGF family is composed of VEGFA, VEGFB, VEGFC, VEGFD and placenta growth factor (PlGF), which are polypeptides with homodimeric structure and are functional related. Three VEGF receptors have been indentified, including VEGFR1, VEGFR2 and VEGFR3, which are also structurally related [295]. Over-expression of VEGF family, which triggered by HIF signaling, promotes neovascularization within solid tumors and function as survival factor of neovessels to inhibit endothelial cells death to further promote angiogenesis in retina [296]. In addition, neuropilin family (NRPs) could form complex with VEGF, for example, NRP1 and NRP2 act as a coreceptor for VEGFR1/2 and VEGFR3, respectively, to regulate angiogenesis [295, 297]. Also, semaphorin (Sema) family and VEGF could be ligands of NRP2 receptors to regulate angiogenesis, respectively, for example, semaphorin 3A (Sema3A) interacting with NRP2 functions as a negative role in regulating physiological and pathological processes of endothelial cell. And studies indicated that Sema3A activation combines with inhibition of VEGF pathway may improve therapeutic efficacy, and thus some small-molecule tyrosine kinase inhibitors, including Axitinib, Cabozantinib, Lenvatinib and Sorafenib etc., are widely used in targeting VEGFR during clinical investigation or treatment (see review by *Bedard, P. L.,* et al.*, 2020*) [298, 299]. Although inhibiting VEGF is a potent and major therapeutic strategy, more efforts need to be invested in understanding mechanisms of multikinases acting on angiogenesis to exploit more efficient anti-angiogenic therapies via epigenetic intervention [298].

### HIF inhibition

Growing evidence have revealed that HIF-1α and HIF-2α inhibitors block tumor growth through a variety of mechanisms [18, 300]. HIF-1α inhibitors have the most types, among which HDAC inhibitors are indirectly involved in epigenetic regulation [136, 301]. Studies have shown that class II HDAC is associated with the stability of HIF-1α and provides a theoretical basis for targeting HIF-1α with HDAC inhibitors [136]. For example, LAQ824 has been reported to promote the polyubiquitination of HIF-1α by unknown mechanisms, thereby inhibiting the function of HIF-α [136]. Due to HIF-1α inhibition, the regulation of HIF-1α by class III HDAC is blocked, thus indirectly blocking the epigenetic regulation of HIF-1α by class III HDAC [176]. In addition, HIF-2α inhibitors, including PT2385 and Vorinostat, inhibit the HIF pathway by interfering with epigenetic mechanisms [302, 303]. Clinically, adjuvant retinoic acid (RA) therapy has a poor response to high-risk neuroblastoma [303]. However, study has shown that 5-Aza-deoxycytidine (AZA) as a DNA-demethylating agent increases the sensitivity of high-risk neuroblastoma to RA, and AZA and RA combined therapy inhibits the growth of high-risk neuroblastoma. This combined treatment induces high levels of transcriptional responses regulated by HIF-2α [303]. After treatment with HIF-2α inhibitor PT2385, the sensitivity of tumor cells to AZA and RA combined therapy decreased, suggesting that HIF-2α is a tumor suppressor in neuroblastoma [303]. In fact, PT2385 indirectly affects DNA methylation involving AZA by inhibiting HIF-2α. More, studies have shown that the expression level of HIF-2α gene EPAS1 in human soft tissue sarcomas (STS) is lower than that of the corresponding normal tissue [302]. Vorinostat, a clinically approved HDAC inhibitor, promotes HIF-2α accumulation, leading to increased tumor growth, which is reversed by HIF-2α deletion [302]. This indicated that Vorinostat mainly affected the transcriptional activity of HIF-2α [302]. These are not the only drugs that target HIF-1α and HIF-2α. These drugs are of great interest in their own right and cannot be ignored.

### Targeting VHL loss for cancer treatment

VHL loss is linked with HIFs over-expression within tumors, especially ccRCC, which observed in clinical trials, and thus small molecule inhibitors that target VHL are widely used in clinical studies [304, 305]. VHL as a tumor suppressor mediates many cellular processes due to its multi-functional role, in which interacted with HIF signaling is the most notably physiological event that VHL binds to Elongin B, Elongin C, Cul2, and Rbx1 proteins forming E3 ligase, leading to HIF-α degradation inhibiting tumor progression [306]. For a long period, the main direction of ccRCC treatment is understanding mechanisms that VHL loss drives tumorigenesis, especially downstream of VHL-HIF pathway, including glutamine metabolic genes, mTOR signaling and lipid metabolism, among others [210]. Glutamine is transformed into glutamate which is subsequently catalyzed into 2-oxo-glutarate (2-OG), and further into lipid via glutaminase enzymatic function in VHL-loss tumor cells. Inhibiting glutaminase blocks the conversion from glutamine to substrates associated with HIF signaling, which may serves as potential therapeutic target [307]. More, mutations in mTOR interdict the PI3K-AKT-mTOR pathway, for example, mutations in PTEN lipid phosphatase and the Tuberous Sclerosis Complex 1/2 (TSC1/2) leads to mTOR dysfunction, which resulting in usage of mTOR inhibitors in clinical treatment [298]. Authentic study noted that VHL-deficient germline resulting in reduced high-density lipoprotein (HDL) cholesterol which is associated with HIF-dependent pathway, and insulin induced gene 2 (INSIG2) is subsequently activated which leads to low levels of fatty acids (FAs) and cholesterol [52]. Similarly, in tumors with VHL loss, HIF-2α activation promotes lipid droplets accumulation followed by elevated expression of both FAs synthesis and FAs absorption-related genes [308]. Cutting off metabolic pathway of HIF signaling, such as lipogenic protein perilipin 2 (PLIN2) and carnitine palmitoyltransferase 1A (CPT1A), may contributes to lipid redistribution, which could be exploited as a therapeutic strategy [304, 309]. More interestingly, OTU deubiquitinase 6B (OTUD6B) as the first reported deubiquitination enzyme is capable of inhibiting pVHL ubiquitination, and the deubiquitination regulation of OTUD6B on pVHL did not depend on its enzyme activity, but via interaction with pVHL to enhance the stability of CBC^VHL^ ubiquitin ligase complex, and reduce the ubiquitination degradation of pVHL by Trp-Asp repeat and suppressors of cytokine signaling box-containing protein 1 (WSB1) and E2-EPF ubiquitin carrier protein (UCP). This provides a new understanding of the mechanisms of the OTU subfamily of deubiquitination enzymes and Cullin-RING ubiquitin ligases [310].

### BET inhibition

Bromodomain and extraterminal domain (BET) family proteins consists of four members, BRD2, BRD3, BRD4 and BRDT, which play an important role as epigenetic readers in gene transcriptional activation [311, 312]. The abnormal activity of the BET protein, which recognizes lysine residues of both histones and non-histones, especially BRD4, is closely associated with cancer progression, making BET a promising therapeutic target [313, 314]. The BET small molecule inhibitors can be used as a promising alternative cancer therapy [315]. Thus, BET inhibitors such as ABBV-075, ABBV-744, BAY 1238097, BMS-986158, CPI-0610, FT-1101, GS-5829, GSK-2820151, GSK-525762, BI-894999, RO-6870810 and OTX-015, have been used to study their efficacy as cancer therapies in clinical trials [312]. Among them, BMS-986158, OTX-015 and GSK-525762 inhibitors are three major clinical stage BET inhibitors [315].

There is limited but growing evidence that BRD4 interacts with HIFs and modulates HIF activities to develop BRD4 as a reliable therapeutic target in multiple cancers, including acute myeloid leukemia, multiple myeloma, and Burkitt’s lymphoma [316, 317]. Studies on BDR4 inhibitors received rapid attention after the inhibitory effects of JQ1 and I-BET762 on BET were reported, in which JQ1 could indirectly regulate the function of HIF through BRD4 [318–320]. Within triple negative breast cancer (TNBC), JQ1 was able to alter the expression activity of 44% of the downstream genes in the HIF pathway, two-thirds of which were down-regulated including CA9, which is related to pH regulation, and VEGF-A, which is related to angiogenesis [318]. Although hypoxia increased the recruitment of BRD4 to CA9 and VEGF-A in tumor cells, the down-regulated CA9 and VEGF-A in tumor cells treated with JQ1 indicated that JQ1 affected the hypoxia response in which BRD4 participated [318]. Recently, Chen and Yin et al. found that NHWD-870, a novel BRD4 inhibitor, effectively inhibited the expression of BRD4 and its target gene HIF-1α [315]. NHWD-870 significantly inhibits tumor growth in a variety of animal tumor models [315]. Mechanistically, NHWD-870 reduces the expression and secretion of macrophage colony stimulating factor 1 (CSF1) in tumor cells by blocking the functional activity of BRD4 and its target gene HIF-1α [315]. Down-regulation of CSF1 weakens the activation degree of ERK1/2 and PI3K/AKT pathways, leading to decreased proliferation and survival of tumor associated macrophages (TAMs) [315]. TAMs rely on tumor-secreted growth factors, such as CSF1, to promote tumor growth and metastasis [321]. This study revealed for the first time that the novel BET inhibitor NHWD-870, which was independently developed, can block the mechanism of tumor-macrophage interaction, providing a theoretical basis for the effective treatment for solid tumors such as melanoma by blocking the new epigenetic target BRD4 in clinical trials [315]. Studies have shown that this inhibitor increases the clinical activity by 5-50 times compared with BMS-986158, OTX-015 and GSK-525762 inhibitors, and can effectively reverse the drug resistance of tumors, which is expected to double the survival time of patients with advanced melanoma [315]. As a first class original new drug in development, NHWD-870 will be the first to carry out phase I clinical trial with melanoma in 2020 [315], which is expected to be a new broad-spectrum anticancer drug, bringing new hope for patients with advanced melanoma, small-cell lung cancer and other diseases. The evidence that BRD4 regulates the activities of HIF-1α and HIF-2α need to be further enriched, and targeting BRD4 is currently considered as a strategy to be developed. Together, BRD4 inhibitor exploitation plays an important potential role in cancer therapy, especially hypoxic tumors.

### Potential treatment based on crosstalk between HIFs signaling and ferroptosis

Ferroptosis is recently discovered iron-dependent cell death fueled by lipid peroxidation, which differs from necrosis and apoptosis [322–324]. Mitochondrial metabolism involved series of substrates consumption, is primary source of ROS via Fenton reaction that ferrous iron mixed with hydrogen peroxide (H_2_O_2_) is equipped with strong oxidizability to oxidize many known intracellular compounds such as carboxylic acids, alcohols and esters into inorganic states with significant oxidation effect [325]. Emerging studies has revealed ROS accumulation in a variety of cells including those within malignancies. Interestingly, it’s reported that hypoxic tumors with HIF-1 over-expression inhibits acyl-CoA dehydrogenases leads to tumor progression via ROS alteration [209]. Over the past 20 years, small-molecule inhibitors, including system x_c_-Inhibitors, GPX4 inhibitors, RTAs, CoQ10 pathway inhibitors, endoperoxides, and iron chelators and sources inhibitors etc., have been applied in ferroptosis-associated diseases [322], and mechanisms behind these inhibition effects may reappear in HIF signaling, which remains under-reported.

Anti-tumor therapy, in addition to established anti-angiogenic therapy, targeting VHL loss and BET inhibition, also has the sustainable treatment based on the potential crosstalk between HIFs and ferroptosis, and targeting CAFs. Epigenetic intervention mediated by small molecule inhibitors are on the way, and higher efficacy may derived from combined joint therapy.

## Conclusion and perspectives

Hypoxia affects tumor progression via various epigenetic modifications, including acetylation, methylation and demethylation and so on, which leads to tumor genomic instability due to tumor and tissue speficity or altered microenvironment. HIF-α activity plays a pivotal role in metabolic reprogramming, for example, special enzymes are activated by HIF-α family to adapt to the obligate metabolic demands, in turn, produced metabolites, including succinate, fumarate, pyruvate, lactate and oxaloacetate etc., affect the HIF proteins stability due to loss-of-function of PHD. Despite of crosstalk between intracellular metabolites production and subsequent metabolic genes activation, EMT is also affected by HIF-α via epigenetic remodelers, notably, HIF-1α promotes P4HA1 and P4HA2 transcriptional activities to further contributes to the formation of collagen, and α-ketoglutarate modulation of P4HA1 boosts the stability of HIF-1α, and further enhance the tumor progression. HIF signaling influences the EMT to promote metastasis, and primary tumors secrete factors which interacted with HIF-α to modify the niche into a survivable system so as to secondary tumor development and progression under hypoxia.

Anti-angiogenetic therapy may be exploited as recognized therapeutic stragedy through epigenetic intervention on VEGF family. Some small molecule tyrosine kinase inhibitors combines with VEGF blockade may improve therapeutic efficacy. However, VEGF signaling interacts with many other genes, it’s critical to fully understand the complex crosstalk and exploit more therapies in face of low curative effects. Besides targeting VHL loss and BET inhibition therapy, given interaction between ferroptosis and HIFs on ROS affects, small-molecule inhibitors of ferroptosis is considered to use in hypoxic tumors treatments. HIF-1α and HIF-2α has been studied a lot, and mounting evidences indicated their important role in the regulation of hypoxic tumor progression, however the pathological role of HIF-3α remains elusive and more efforts should be placed in understanding its pathological function. In fact, Ras/raf/MAPK, NF-κB, SREBP, Jak/Stat and Notch pathways are also play important roles in tumorigenesis, the interactions between them and HIF signaling may be more complex and need to be further studied.

Up to now, given the role of HIF signaling in hypoxic tumor progression, it’s of great potential importance that targeting HIF-α directly or inhibiting the downstream genes in the crosstalk instead.

## Data Availability

Not applicable.

## Abbreviations

HIFs: Hypoxia-inducible factors
PHD2: Prolyl hydroxylase domain protein 2
VHL: Hippel-Lindau tumor suppressor
ARNT: Aryl hydrocarbon receptor nuclear translocator
lncRNAs: long non-coding RNAs
TIMP2: Tissue inhibitor of metalloproteinases 2
HCC: Hepatocellular carcinoma
ODD: Oxygen-dependent degradation
OXPHOS: Oxidative phosphorylation
AMPK: AMP-activated protein kinase
ROS: Reactive oxygen species
NF-κB: Nuclear factor kappa-B
TNF-α: Tumor necrosis factor alpha
TAK: Tat-associated kinase complex catalytic subunit
IKK: I-kappaB kinase
CDK6: Cyclin-dependent kinase 6
PI3K: Phosphoinositide 3-kinase
PDK: Pyruvate dehydrogenase kinase
PKB: Protein kinase B
mTOR: Rapamycin
FoxO1: Forkhead box protein O1
DLL4: Delta like canonical Notch ligand 4
Notch: Neurogenic locus notch homolog
SAM: S-adenosine methionine
5mC: 5-methylcytosine
CA9: Carbonic anhydrase 9
TET: Ten-eleven-translocation 5-methylcytosine dioxygenases
2-OGDDs: 2-oxoglutarate-dependent dioxygenases
CRS: Chronic restraint stress
SI-DhMLs: Hydroxymethylated loci
ChIP-seq: Chromatin immunoprecipitation sequencing
HRE: Hypoxic response element
m^6^A: N6-methyladenosine
GLP: G9a-like protein
Ehmt2: Euchromatic histone lysine methyltransferase 2
VEGF: Vascular endothelial growth factor
JMJD: Jumonji domain
JmjC: Jumonji C
2OG: 2-oxoglutarate
FAD: Flavin adenine dinucleotide
PTC: Papillary thyroid carcinoma
TIP60: TAT-interactive protein 60
CBP: Cyclic AMP response element-binding protein
PCAF: CBP-associated factor
DANCR: Differentiation antagonizing non-protein coding RNA
MIIP: Migration and invasion inhibitory protein
α-KG: α-ketoglutarate
FBP1: Fructose-1,6-bisphosphatase
GLUT: Glucose transporter
HK: Hexokinase
PYG: Phosphorylase
PFKFB: 6-phosphofructo-2-kinase/fructose-2,6-bisphosphatase
PDH: Pyruvate dehydrogenase
FA: Fatty acid
FASN: Fatty acid synthase
ACC: Acetyl-CoA carboxylase
FAO: Fatty acid oxidation
PTEN: Phosphatase and tensin homolog
HMGCS2: 3-hydroxy-3-methylglutaryl CoA synthase 2
IDH2: Isocitrate dehydrogenase 2
GDH: Glutamate dehydrogenase
HOXA9: Homeobox A9
CML: Chronic myelogenous leukemia
IPO7: Importin 7
ECM: Extracellular matrix
5meC: 5-methylcytosine
MDSCs: Myeloid-derived suppressor cells
regDCs: regulatory dendritic cells
TAMs: Tumor-associated macrophages
NMT3A: DNA methyltransferase 3A
EZH2: Enhancer of zeste homolog 2
Mcrc: Metastatic colorectal cancer
HA: Hyaluronic acid
LOXL2: Lysyl oxidase-like protein 2
L1CAM: L1 cell adhesion molecule
CYTIP: Cytohesin 1 interacting protein
PRC2: Polycomb repressive complex 2
WDR5: WD repeat containing protein 5
HMT: Histone methyltransferase
TUG1: Taurine upregulated gene 1
CAFs: Cancer-associated fibroblasts
UBE2CP3: Ubiquitin conjugated enzyme E2C pseudogene 3
HUVEC: Human umbilical vein endothelial cell
BET: Bromodomain extralterminal proteins
PlGF: Placenta growth factor
NRPs: Neuropilin family
Sema3A: Semaphorin 3A
STS: Soft tissue sarcomas
TSC1/2: Tuberous Sclerosis Complex 1/2
INSIG2: Insulin induced gene 2
PLIN2: Lipogenic protein perilipin 2
CPT1A: Carnitine palmitoyltransferase 1A
OTUD6B: OTU deubiquitinase 6B
WSB1: Trp-Asp repeat and suppressors of cytokine signaling box-containing protein 1
UCP: E2-EPF ubiquitin carrier protein
CSF1: Colony stimulating factor 1
H_2_O_2_: Hydrogen peroxide
